# Global prevalence and epidemiology of *Strongyloides stercoralis* in dogs: a systematic review and meta-analysis

**DOI:** 10.1186/s13071-021-05135-0

**Published:** 2022-01-10

**Authors:** Aida Vafae Eslahi, Sima Hashemipour, Meysam Olfatifar, Elham Houshmand, Elham Hajialilo, Razzagh Mahmoudi, Milad Badri, Jennifer K. Ketzis

**Affiliations:** 1 Medical Microbiology Research Center, Qazvin University of Medical Sciences, Qazvin, Iran; 2 Metabolic Diseases Research Center, Research Institute for Prevention of Non-Communicable Diseases, Qazvin University of Medical Sciences, Qazvin, Iran; 3 Department of Parasitology, Faculty of Veterinary Medicine, Rasht Branch, Islamic Azad University, Guilan, Iran; 4 Department of Parasitology and Mycology, Qazvin University of Medical Sciences, Qazvin, Iran; 5 Student Research Committee, Qazvin University of Medical Sciences, Qazvin, Iran; 6 Ross University School of Veterinary Medicine, Basseterre, West Indies St. Kitts and Nevis

**Keywords:** *Strongyloides stercoralis*, Canine, Neglected tropical disease, Soil transmitted helminth, Systematic review

## Abstract

**Background:**

*Strongyloides stercoralis*, a soil-transmitted helminth, occurs in humans, non-human primates, dogs, cats and wild canids. The zoonotic potential between these hosts is not well understood with data available on prevalence primarily focused on humans. To increase knowledge on prevalence, this review and meta-analysis was performed to estimate the global status of *S. stercoralis* infections in dogs.

**Methods:**

Following the PRISMA guidelines, online literature published prior to November 2020 was obtained from multiple databases (Science Direct, Web of Science, PubMed, Scopus and Google Scholar). Prevalence was calculated on a global and country level, by country income and climate, and in stray/animal shelter dogs versus owned dogs. Statistical analyses were conducted using R-software (version 3.6.1).

**Results:**

From 9428 articles, 61 met the inclusion criteria. The estimated pooled global prevalence of *S. stercoralis* in dogs was 6% (95% CI 3–9%). Infection was found to be the most prevalent in low-income countries with pooled prevalence of 22% (95% CI 10–36%). The highest pooled prevalence of *S. stercoralis* in dogs was related to regions with average temperature of 10–20 °C (6%; 95% CI 3–11%), an annual rainfall of 1001–1500 mm (9%; 95% CI 4–15%) and humidity of 40–75% (8%; 95% CI 4–13%). Prevalence was higher in stray and shelter dogs (11%; 95% CI 1–26%) than in owned dogs (3%; 95% CI 1–7%).

**Conclusions:**

As with *S. stercoralis* in humans, higher prevalence in dogs is found in subtropical and tropical regions and lower-income countries, locations which also can have high dog populations. While this study presents the first estimated global prevalence of *S. stercoralis* in dogs, it is potentially an underestimation with 15 of 61 studies relying on diagnostic methods of lower sensitivity and a paucity of data from most locations. Standardized protocols (e.g. quantity of feces and number of samples for a Baermann) in future studies could improve reliability of results. More prevalence studies and raising veterinary awareness of *S. stercoralis* are needed for a One Health approach to protect humans and dogs from the impact of the infection.

**Graphical Abstract:**

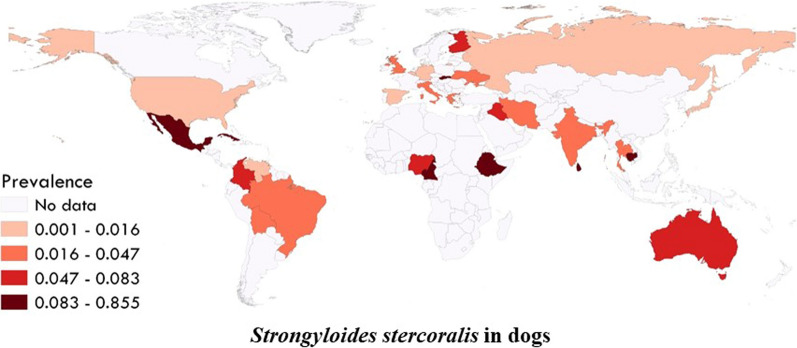

**Supplementary Information:**

The online version contains supplementary material available at 10.1186/s13071-021-05135-0.

## Background

A quarter of the world’s population is impacted by helminthic infections that cause substantial rates of diseases and/or disabilities. Many of these helminths are zoonotic with carnivores, particularly dogs and cats, responsible for transmission of nearly 43% of the zoonotic pathogens [1–4]. One of these zoonotic pathogens is *Strongyloides stercoralis*, a soil-transmitted helminth that affects 100–370 million people globally and is classified as a neglected tropical disease [5, 6]. The main manifestations of *S. stercoralis* infection are gastrointestinal and cutaneous signs. However, *S. stercoralis* infections can be asymptomatic but, in the other extreme, can cause severe pulmonary pathology with auto- and hyperinfection [7–9]. The life cycle of *S*. *stercoralis* involves homogonic and heterogonic stages. In the homogonic cycle, only females exist in the host and eggs are produced via parthenogenesis. First-stage larvae (L1) and occasionally in some hosts eggs containing L1 are excreted via the host’s feces into the environment where the heterogonic cycle occurs. Male and female larvae molt into free-living adult worms through four larval stages with female larvae also being able to molt into infectious third-stage larvae (L3) with no further development until entering a host [8]. The most common techniques to detect larvae of *S. stercoralis* in human feces are direct smear, Kato-Katz, flotation, sedimentation, Baermann and Koga agar plate culture with the latter three being the more sensitive for larvae. Indirect fluorescent antibody tests (IFATs), enzyme-linked immunosorbent assays (ELISAs) and molecular methods also can be used for diagnosis but are more frequently used in research versus clinical settings [10–12]. The primary treatment for *S. stercoralis* infection in humans is ivermectin [9].

*Strongyloides stercoralis* also is capable of infecting canids and a range of other vertebrate hosts such as felids and non-human primates [6, 13]. Canine *S. stercoralis* infection occurs most frequently in puppies and young dogs under 1 year of age and in puppies living in breeding kennels with poor sanitary conditions during hot and humid seasons [14–18]. Infection in dogs can be asymptomatic, but also can be life-threatening with clinical signs ranging from diarrhea and malabsorption to bronchopneumonia. Extraintestinal disseminations such as the nasal cavities, lungs, stomach and cranial cavity associated with severe clinical signs have been documented in immunocompromised canids as in humans (e.g. due to other pre-existing conditions or administration of immunosuppressive medicines) [19–21]. The methods for detection of *S. stercoralis* in dog feces are the same as those in humans with treatments including not only ivermectin but also fenbendazole, albendazole and selamectin although no products are registered for this use in dogs [22].

Dogs and humans share certain *S. stercoralis* genotypes. Although there are few reports of transmission from dogs to humans, experimental infections illustrate that *S. stercoralis* from human origin can infect dogs, suggesting dogs can be a reservoir for human infection [6, 13, 23, 24]. While there have been recent studies estimating regional and global prevalence of *Strongyloides* for humans and associated risk factors [5, 7, 12, 25], these data are not available for infections in dogs. In a one health context, better knowledge on the prevalence of *S. stercoralis* infection in dogs and the risk of zoonotic transmission to humans is needed. Therefore, this review and meta-analysis aimed to estimate the global prevalence of *S. stercoralis* in dogs and assess some variables that might influence prevalence.

## Methods

### Search strategy

This systematic review and meta-analysis followed PRISMA guidelines (http://www.prisma-statement.org/). A systematic literature search was carried out on multiple general science databases to identify all publications reporting *S. stercoralis* in dogs across the world published prior to November 2020. Science Direct, Web of Science, PubMed, Scopus and Google Scholar were explored using the following search terms: *Strongyloides stercoralis*, *S. stercoralis*, strongyloidiasis, dogs, puppies, gastrointestinal helminths, soil-transmitted helminths, worldwide and prevalence using AND and/or OR Boolean operators. Two independent authors involved in the search evaluated titles and abstracts and reviewed the full-text papers. After removing duplicates and irrelevant records, reference lists of full texts were examined for potential eligibility of citations not found in the database search.

### Inclusion and exclusion criteria and data extracted

Literature was eligible for inclusion if it met the following priori criteria: (1) peer-reviewed articles containing original data, (2) cross-sectional studies reporting the prevalence of strongyloidiasis in dogs, (3) accessible full text and abstract and (4) numerator and denominator data available to confirm prevalence values. Literature that did not satisfy the aforementioned criteria, such as review articles with no original data, letters, editorials, articles with fecal material collected from the ground and lack of clarity about whether there were repeated samples and articles with ambiguous/undetermined conclusions, were excluded. Articles that reported *S. stercoralis* in humans, animals other than dogs and soil were excluded.

Using a Microsoft Excel^®^ spreadsheet, the following information was retrieved from the included articles: first author name, year of publication, country where the study was conducted, continent, sample size and number of positive cases, diagnostic method(s) used, income level (https://datahelpdesk.worldbank.org/knowledgebase/articles/906519-world-bank-country-and-lending-groups), humidity (https://www.timeanddate.com/weather/iran/tehran/climate), annual rainfall (https://en.climate-data.org/), average temperature (https://en.climate-data.org/), latitude, (https://www.geodatos.net/en/coordinates/) and climate (https://www.britannica.com/science/Koppen-climate-classification). Antibody seroprevalence studies were excluded, since they cannot confirm current infection. Experimental antigen methods also were excluded. In studies where more than one method was used to analyze a single sample (e.g. Baermann and flotation), the total number of positive samples was determined and used in the analysis.

### Quality assessment

The Newcastle-Ottawa Scale was used to assess the quality of the included articles [26]. A maximum score of 9 was assigned to each article based on subject selection (0–4 points), comparability of subjects (0–2 points) and exposure (0–3 points). A total score of 0–3, 4–6 and 7–9 points was considered poor, moderate and high quality, respectively [27–29].

### Data synthesis and statistical analysis

The pooled prevalence of *S. stercoralis* in dogs reported globally and by continent was calculated with 95% confidence intervals (CIs). In addition, prevalence for stray and shelter dogs was compared to that of owned dogs with owned dogs defined as household owned, those in pet stores and breeding dogs but excluding those with only breeding kennel data. Sub-group analysis included country income level, humidity, annual rainfall, average temperature and latitude. The probability of publication bias was surveyed using Egger’s regression test and Begg’s test. A meta-regression analysis was conducted to evaluate the impact of the year of publication on prevalence. All statistical analyses were performed using the meta-package in *R* (version 3.6.1). The pooled prevalence estimates were computed using the alpha method for the random-effects model, based on the inverse variance approach for measuring weight. Cochrane’s *Q* test and inconsistency index (*I*^2^ statistics) were used to assess the magnitude of heterogeneity among included studies, with *I*^2^ values of  < 25%, 25–75% and  < 75% considered as low, moderate and high heterogeneity, respectively. A *P* value < 0.05 was considered statistically significant.

## Results

### Literature search, selection and data extraction

**Fig. 1:**
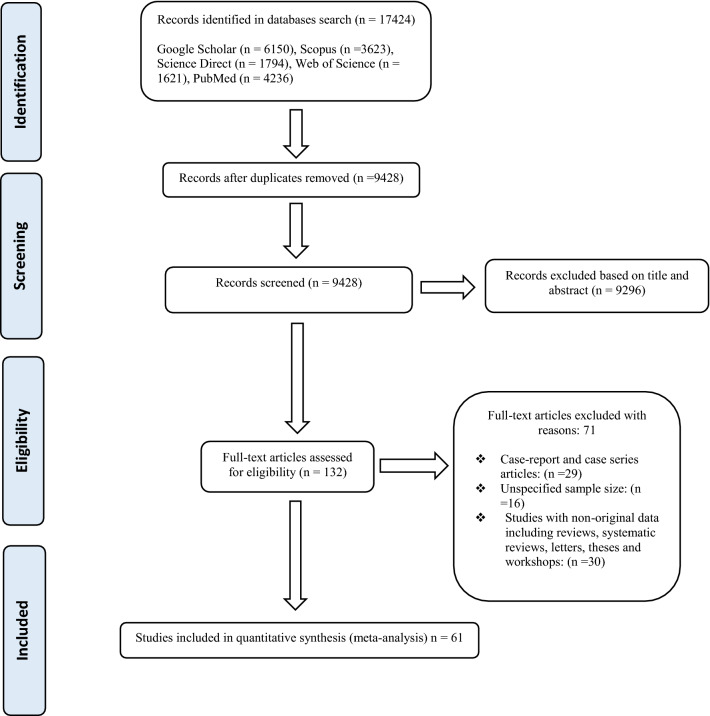
Flow diagram representing the selection of studies for inclusion in the systematic review and meta-analysis of the global prevalence of *Strongyloides* in dogs

Our systematic search yielded a total of 9428 publications. One hundred thirty-two full-text articles were chosen for eligibility assessment. There were 16 studies with an unspecified sample size and 55 studies with no original data, including reviews, case reports and case series, letters, theses and workshops. Finally, 61 studies were included in the meta-analysis based on critical appraisal criteria (Fig. 1; Table 1 with full list of references in Additional file 1: References S1). The included studies utilized parasitology techniques comprising microscopic methods (flotation with and without concentration, sedimentation, Baermann, Kato-Katz and other direct smear methods, and necropsy), culture methods (Harada-Mori and agar plate culture), molecular techniques [conventional polymerase chain reactions (PCR) and real-time PCR] and serological methods (IFAT and ELISA). Of the 61 articles, 15 used only direct smears and/or flotation with most studies using sedimentation and/or Baermann often in combination with flotation or direct smears. Four studies used culture, three of which were combined with other methods. Only eight of the included articles used diagnostic methods other than microscopy and culture: three used immunological methods in addition to microscopic or culture methods; one used serology, PCR, microscopic and culture methods; one used PCR and microscopic methods; and one used only PCR methods.

**Table 1:** Main characteristics of the included studies reporting the prevalence of *Strongyloides stercoralis* in dogs

No.	First author	Publication year	Country	Continent	Diagnostic methods used	Quality assessment based on Newcastle–Ottawa Scale
1	Rizzo and Ricciardi	1978	Italy	Europe	Unclear	5
2	Ugochukwu and Ejimadu	1985	Nigeria	Africa	Saturated solution flotation, formalin-ether sedimentation	5
3	Tarish et al.	1986	Iraq	Asia	Necropsy	6
4	Stehr-Green et al.	1987	USA	North America	Formalin-ethyl acetate sedimentation	9
5	Epe et al.	1993	Germany	Europe	Coproscopical examinations	6
6	Bugg et al.	1999	Australia	Oceania	Sedimentation, zinc sulfate (ZnSO_4_) flotation	7
7	Itoh et al.	2003	Japan	Asia	Coproscopical examination	7
8	Anosike et al.	2004	Nigeria	Africa	Direct smear, concentration methods	5
9	Asano et al.	2004	Japan	Asia	Direct smear, saturated salt (NaCl) flotation, ZnSO_4_ flotation, sucrose flotation	8
10	Ramirez-Barrios et al.	2004	Venezuela	South America	NaCl flotation	8
11	Komtangi et al.	2005	Cameroon	Africa	McMaster	5
12	Júnior et al.	2006	Brazil	South America	Baermann, sedimentation, ELISA, IFAT	9
13	Gonçalves et al.	2007	Brazil	South America	Baermann, sedimentation, ELISA, IFAT	8
14	Lorenzini et al.	2007	Brazil	South America	Saturated NaCl flotation, ZnSO_4_ flotation	6
15	Papazahariadou et al.	2007	Greece	Europe	Teleman’s sedimentation	8
16	Dillard et al.	2007	Finland	Europe	Baermann	7
17	Ugbomoiko et al.	2008	Nigeria	Africa	Kato-Katz thick smear	7
18	Das et al.	2009	India	Asia	Unclear	5
19	Claerebout et al.	2009	Belgium	Europe	Sucrose flotation	8
20	Gates and Nolan	2009	USA	North America	ZnSO_4_ flotation, formalin-ethyl acetate sedimentation	8
21	Itoh et al.	2009	Japan	Asia	Formalin-ethyl acetate sedimentation	7
22	Takano et al.	2009	Japan	Asia	Direct smear, agar plate culture (APC)	7
23	Leelayoova et al.	2009	Thailand	Asia	Direct smear, formalin-ethyl acetate concentration	6
24	Razmi	2009	Iran	Asia	Mini Parasep^®^ SF (sedimentation)	7
25	Mariana et al.	2010	Bolivia	South America	Willis-Malloy flotation	5
26	Zewdu et al.	2010	Ethiopia	Africa	Necropsy	6
27	Awoke et al.	2011	Ethiopia	Africa	Direct smear, flotation	5
28	Jones et al.	2011	Ethiopia	Africa	Necropsy	5
29	Itoh et al.	2011	Japan	Asia	Formalin-ethyl acetate sedimentation	8
30	Itoh et al.	2011	Japan	Asia	Formalin-ethyl acetate sedimentation	7
31	Paulos et al.	2012	Ethiopia	Africa	Sedimentation and flotation	5
32	Martins et al.	2012	Brazil	South America	PARATEST^®^ Diagnostek (sedimentation)	7
33	Getahun and Addis	2012	Ethiopia	Africa	Sedimentation and NaCl flotation	6
34	Mircean et al.	2012	Romania	Europe	NaCl flotation	8
35	Mekbib et al.	2013	Ethiopia	Africa	Direct smear, flotation and sedimentation	5
36	G/selasie et al.	2013	Ethiopia	Africa	McMaster, sedimentation	5
37	Abere et al.	2013	Ethiopia	Africa	Direct smear, sedimentation and NaCl flotation	6
38	Perera et al.	2013	Sri Lanka	Asia	NaCl flotation, Sheather’s sucrose flotation, direct smear	6
39	Riggio et al.	2013	Italy	Europe	Flotation, Baermann	8
40	Ortuno et al.	2014	Spain	Europe	ZnSO_4_ flotation	7
41	Alvarado-Esquivel et al.	2015	Mexico	North America	Sheather’s and ZnSO_4_ flotation	8
42	Puebla et al.	2015	Cuba	North America	Direct smear, formalin ethyl acetate sedimentation, Kato-Katz smear, Willy-Malloy flotation	5
43	Elom et al.	2015	Nigeria	Africa	NaCl and ZnSO_4_ floatation Formol ether sedimentation	5
44	Hadi and Faraj	2016	Iraq	Asia	Direct smear, potassium dichromate K_2_Cr_2_O_4_ film, NaCl flotation, Formalin-ether sedimentation	5
45	Wright et al.	2016	UK	Europe	FLOTAC technique	7
46	Ferreira et al.	2016	Brazil	South America	Sucrose and NaCl flotation, water-ether sedimentation	7
47	Pumidonming et al.	2016	Thailand	Asia	Flotation, formalin-ethyl acetate sedimentation	7
48	Strkolcova et al.	2017	Slovakia	Europe	Flotation, Baermann, APC, ELISA	7
49	Paradies et al.	2017	Italy	Europe	Direct smear, Baermann, necropsy	8
50	Mircean et al.	2017	Romania	Europe	NaCl flotation, sedimentation	8
51	Jaleta et al.	2017	Cambodia	Asia	Baermann, Kato-Katz	9
52	Sauda et al.	2018	Italy	Europe	Flotation, Baermann	8
53	García et al.	2018	Venezuela	South America	NaCl flotation	5
54	Hurtado and Forero	2019	Colombia	Asia	Formalin-gasoline concentration	5
55	Iatta et al.	2019	Italy	Europe	Direct smear, Baermann, APC, IFAT, RT-PCR	8
56	Sanchez-Thevenet et al.	2019	Spain	Europe	Modified Ritchie formalin-ether, Sheather’s sugar flotation, RT-PCR	8
57	Kurnosova et al.	2019	Russia	Europe	NaCl and ammonium nitrate flotations	7
58	Sanpool et al.	2020	Thailand	Asia	APC	7
59	Beknazarova et al.	2020	Australia	Oceania	qPCR, RT-PCR	8
60	Dashchenko et al.	2020	Ukraine	Europe	Direct smear, Baermann, modified string test	6
61	Nagamori et al.	2020	USA	North America	Direct smear, flotation, sedimentation, Baermann	8

### Pooled prevalence

**Fig. 2:**
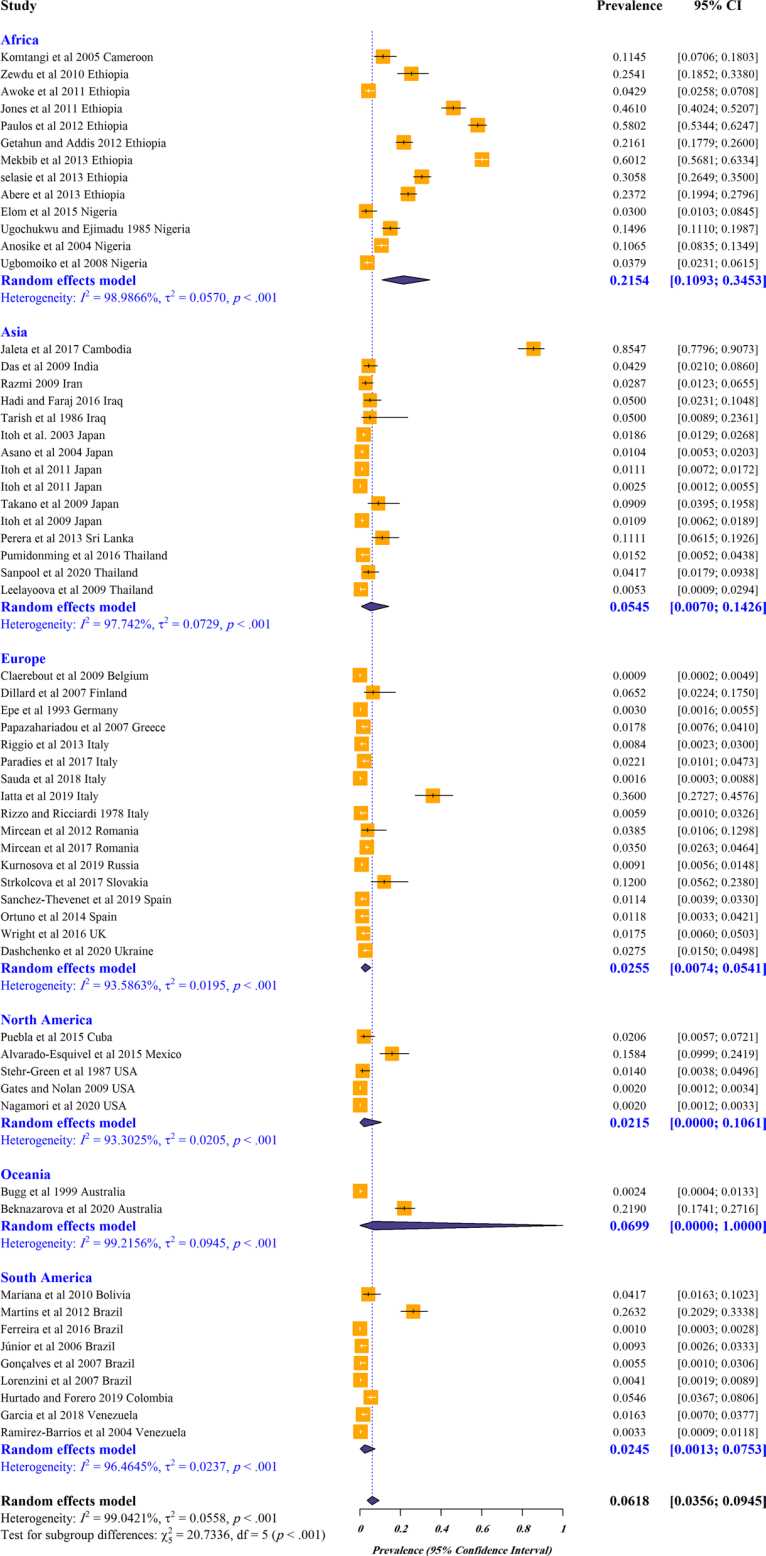
Forest plots for random-effects meta-analysis of the global prevalence of *Strongyloides stercoralis* in dogs based on continent

**Fig. 3:**
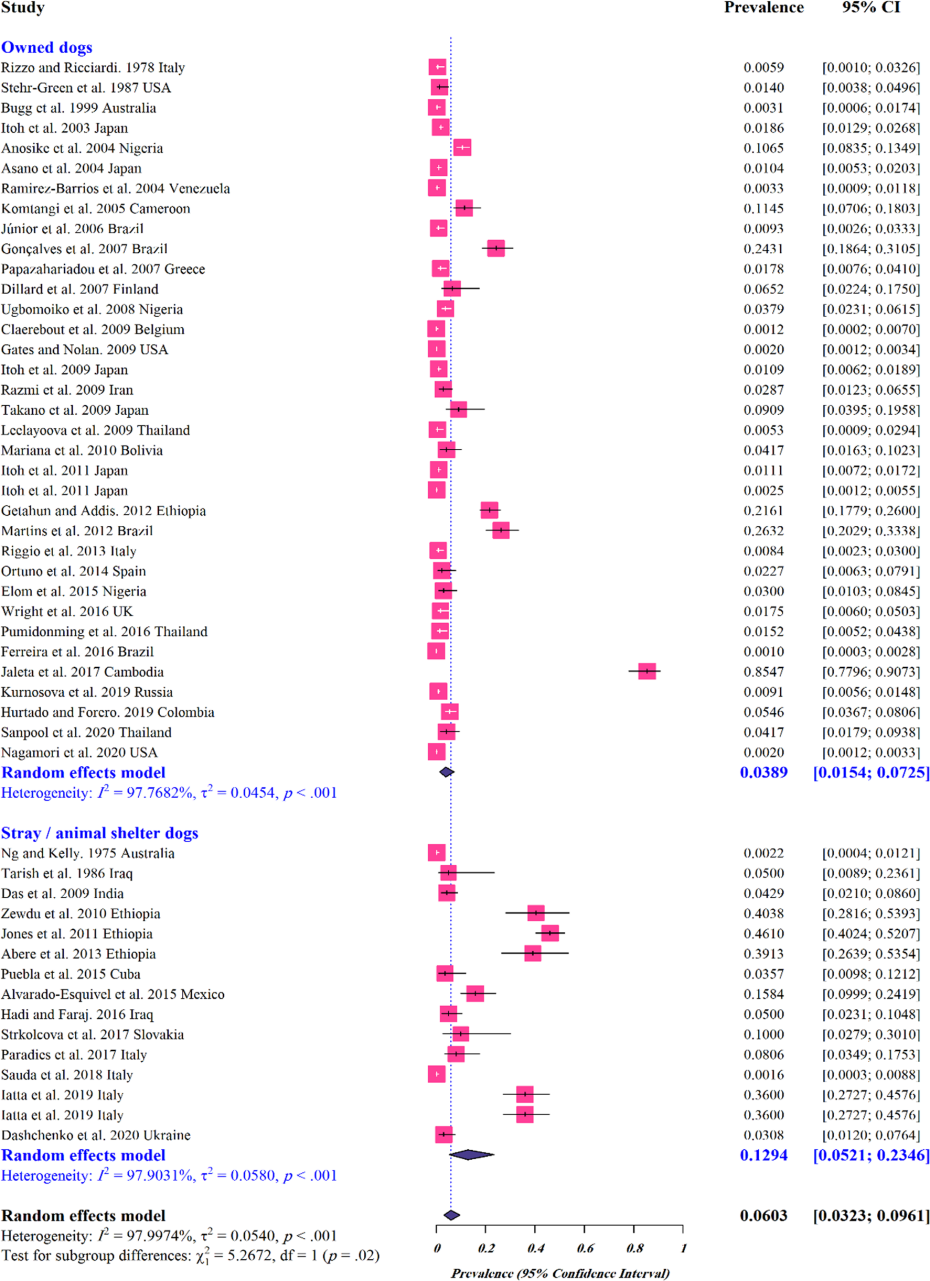
Forest plots for random-effects meta-analysis of the global prevalence of *Strongyloides stercoralis* in owned and stray/shelter dogs

**Fig. 4:**
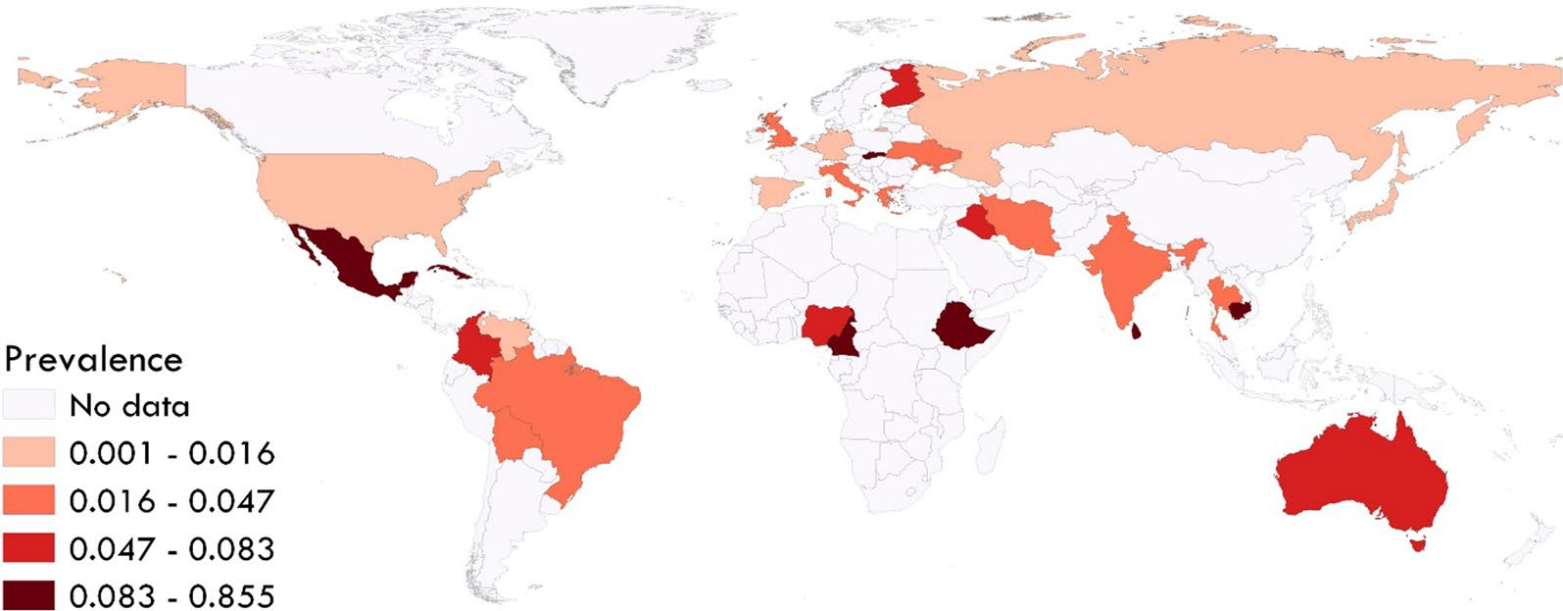
Global prevalence of *Strongyloides stercoralis* in dogs based on included studies

The estimated pooled global prevalence for *S. stercoralis* in dogs was 6% (95% CI 3–9%) with a higher estimated pooled prevalence in stray/shelter dogs (11%, 95% CI 1–26%) than in owned dogs (3%, 95% CI 1–7%) (Figs. 2;  3). Based on the manuscripts included in the analysis, *S. stercoralis* infection in dogs has been documented in 29 countries (Fig. 4; Additional file 2: Figure S1). The pooled prevalence on different continents ranged from 21 to 2%, with 21% (95% CI 10–34%) in Africa, 6% (95% CI 0–100%) in Oceania, 5% (95% CI 0–14%) in Asia, 2% (95% CI 0–10%) in North America, 2% (95% CI 0–7%) in South America and 2% (95% CI 0–5%) in Europe (Fig. 2; Additional file 3: Table S1).

The largest number of studies was conducted in Ethiopia (8 studies), followed by Japan (6 studies). Analyses based on countries showed that Cambodia had the highest pooled prevalence (85%, 95% CI 78–91%) (Additional file 2: Figure S1). The estimated pooled prevalence based on country-level income groups ranged from 22 to 2%, with the highest rate in low-income countries (22%, 95% CI 10–36%) (Additional file 3: Table S1).

Our analyses revealed that regions with average temperatures of 10–20 °C had the highest prevalence of *S*. *stercoralis* (6%, 95% CI 3–11%) (Additional file 3: Table S1). Furthermore, the infection was more prevalent in regions with humidity of 40–75% (8%, 95% CI 4–13%), annual rainfall of 1001–1500 mm (9%, 95% CI 4–15%) and a tropical wet and dry climate (12%, 95% CI 5–21%). In addition, we found that the highest prevalence rate was at a latitude of 1°–25° (11%, 95% CI 5–19%).

### Publication bias

**Fig. 5:**
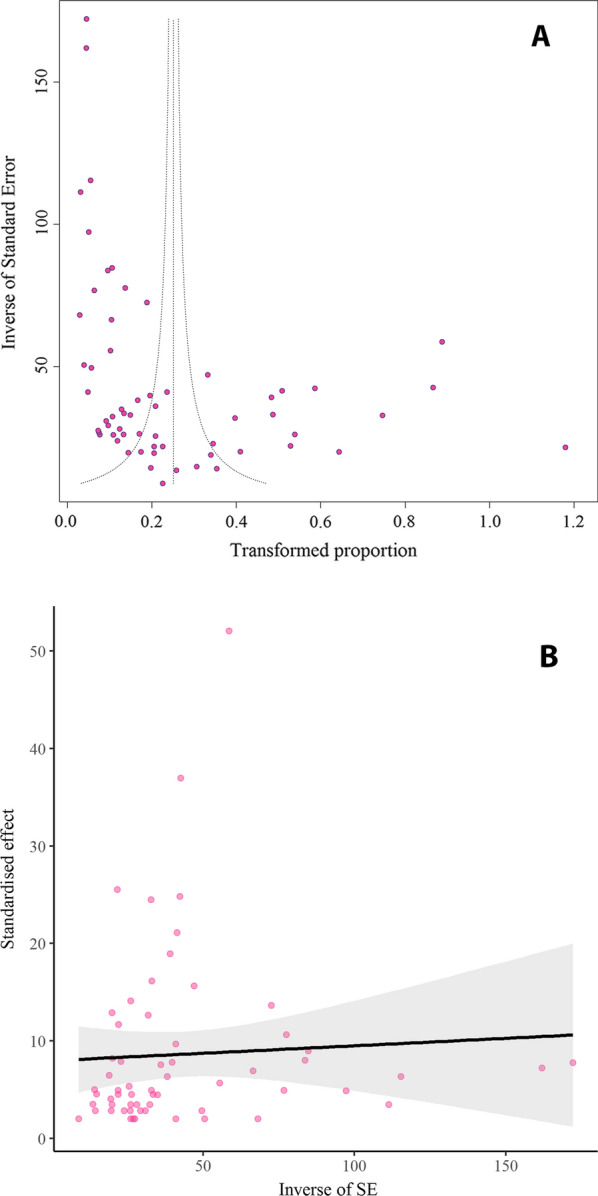
Egger’s funnel plot (**A**) and Begg’s funnel plot (**B**) to assess publication bias in included studies. (Colored circles represent each study. The middle line is the effect size and the other two lines are the corresponding confidence ranges)

**Fig. 6:**
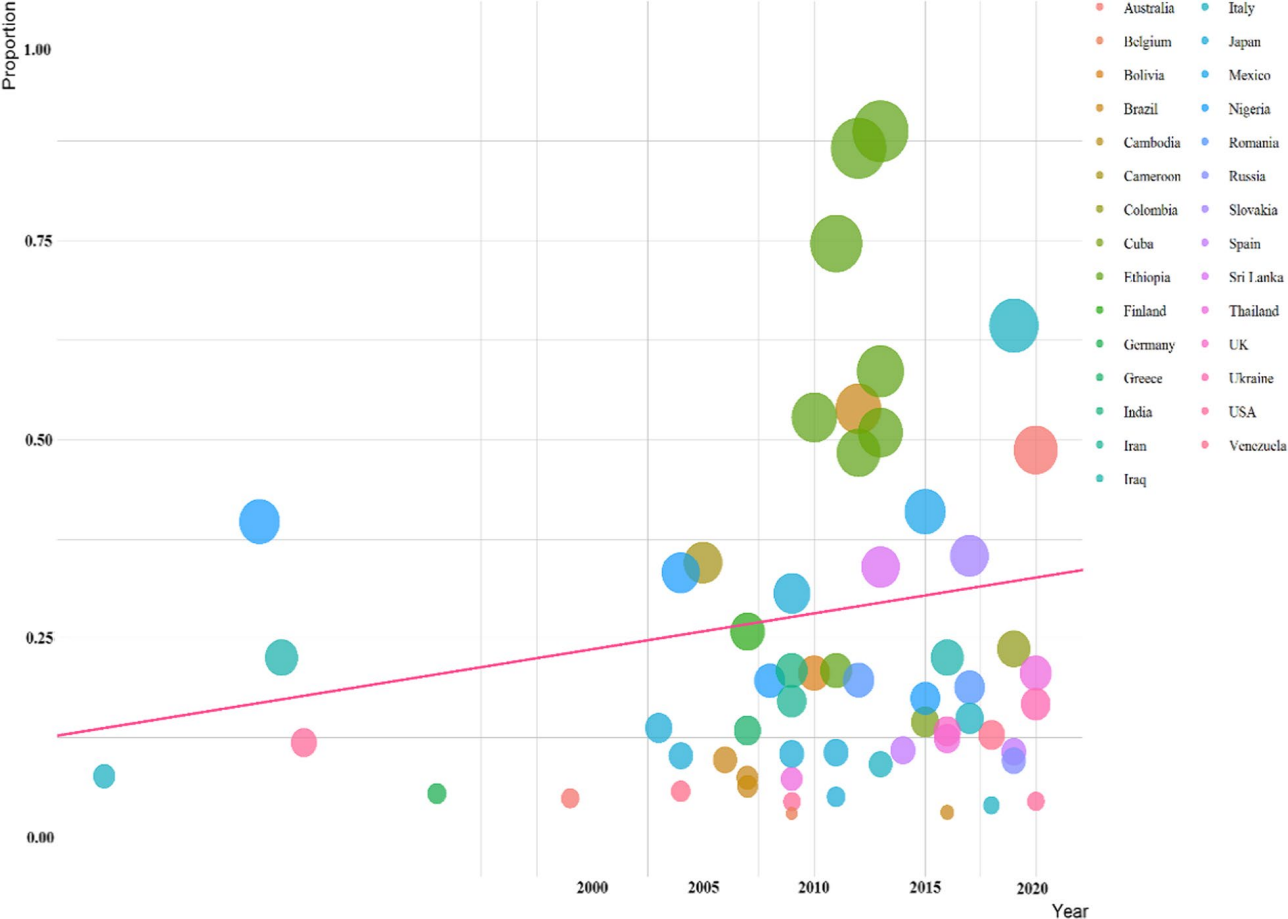
A meta-regression graph for the prevalence of *Strongyloides* in dogs based on the year of publication. (The pink line is the regression line, which was plotted based on the intercept and the slope of the regression model. The different color bubbles represent the countries under study and their sizes indicate the effect size of each study)

As demonstrated by funnel plot asymmetry, a highly significant publication bias was observed in our study using Egger’s test (*t*  = 4.12, *P*  = 0.0001) and Begg’s test (*P * = 0.001) (Fig. 5A, B). Meta-regression analysis demonstrated that there was a significant heterogeneity between studies regarding the year of publication (regression slope  = 0.0055, *P*  = 0.0116) (Fig. 6). Evaluation of study quality revealed that, among 61 studies, 36 had a total score of 7–9 points (high quality) and 25 had a total score of 4–6 points (moderate quality). No included studies were considered poor quality (Table 1).

## Discussion

Dogs are among the most popular companion animals with a considerable positive impact on the psychological and physiological conditions of their owners [30, 31]. This close relationship with dogs, however, can pose certain risks with the potential of dogs transmitting a broad range of zoonotic pathogens, including viruses, bacteria, parasites and fungi [32–34]. Regarding *S. stercoralis*, some genotypes are dog specific while others can infect dogs and humans; hence, dogs could be reservoirs of zoonotic *S. stercoralis* [6, 13, 35]. In this review and meta-analysis, we estimated the global prevalence of *S*. *stercoralis* in dogs. Our findings show *S. stercoralis* in dogs being documented in 29 countries and that infections are not limited to dogs in tropical and subtropical regions, although these regions have the higher prevalence as is the general case for human infections [12].

Factors that potentially contribute to the higher prevalence in tropical regions include temperature and humidity, country income and presence of stray dogs. Similar to prior studies, the results of the meta-analysis herein presented indicate that climate conditions play a key role in the prevalence of *S. stercoralis* infection with the highest pooled prevalence in areas with a tropical wet and dry climate [12]. The higher humidity and temperature make favorable conditions for survival of heterogonic stages of *S. stercoralis* [36, 37]. While the climate plays a key role in prevalence, several studies have confirmed higher rates of helminthic infections in humans in low-income countries, attributed to sanitary conditions and limited access to health care [38–40]. This is similar to our results, with the highest prevalence of *S. stercoralis* in dogs in low-income countries. In these countries, access to veterinary care, specifically access to or affordability of anthelmintics, might be limited, contributing to the higher prevalence. Also, in low-income countries, the number of stray dogs can be high with the prevalence of *S. stercoralis* infection being greater than that in owned dogs, based on the data from the meta-analysis herein presented [41, 42].

Cambodia, where the highest prevalence was found in our analysis, serves as a potential example of the interaction of climate, income and free-roaming (individually or community owned but not contained) or stray dogs. Cambodia is a lower middle-income country with a tropical climate and limited accessibility to improved sanitation services as well as safe drinking water [43]. In the human population, *S. stercoralis* is a public health concern with prevalence being > 40%, one of the higher levels in human populations that have been found [44–46]. Lastly, many dogs in the country are free-roaming or stray [47]. These potential interactions support the need for a One Health approach to addressing *Strongyloides* infections in humans and dogs.

There is no gold standard method for detecting *S. stercoralis* in dogs (or people), and the current techniques have limited sensitivity due to the low burden of parasites and intermittent larval shedding [21, 48]. The most commonly used methods for the diagnosis of *S. stercoralis *infection in humans are direct smear and Kato-Katz, both of which have low sensitivity [12, 46]. Sedimentation, the Baermann method and agar plate culture have higher sensitivity, but they are inconvenient, time-consuming and still underestimate infections [12, 49]. While these latter methods were used in most of the studies included in the meta-analysis, technical details were inconsistent and their implementation varied. For example, with the Baermann the quantity of fecal material and the number of samples analyzed (e.g. one or three from consecutive days) were not standardized across studies, thus resulting in varied sensitivity of the method across studies. In some of the included articles, the focus was on general parasite prevalence with flotation and smears used for fecal analysis, standard screening methods for parasites in dogs but methods with low sensitivity for *S. stercoralis*, potentially resulting in an underestimation of prevalence. Interpretation of results from serological and molecular-based techniques, which were used in a few of the included articles, must be made with caution because of the possibility of false-positive and/or -negative results [21]. Given the high variation in how each diagnostic method was used in the studies, sensitivity ranges could not be assigned with confidence; hence, in the meta-analysis herein presented, prevalence was not adjusted based on the diagnostic method used with the resulting global prevalence likely underestimated.

In a clinical setting, *S. stercoralis* infections in dogs also are likely to be underestimated or overlooked because of the challenge of differentiating *S*. *stercoralis* larvae from other larvae that can occur in feces [i.e. *Angiostrongylus vasorum*, *Crenosoma vulpis*, *Filaroides* (*Oslerus*) *osleri*, *Filaroides hirti* and *Filaroides milksi*] [21]. Also, feces must be directly collected from the rectum or collected from the ground immediately after defecation to prevent fecal contamination with the larvae of free-living nematodes.

Most of the studies included in the meta-analysis were from tropical and subtropical regions, biasing the result towards higher prevalence in these regions. In other regions, within specific dog populations, prevalence might be higher than indicated in the meta-analysis with studies targeting *S. stercoralis* in susceptible dog populations (e.g. kennels, strays and shelter dogs) having prevalence similar to that seen in tropical and subtropical regions [16, 19, 50]. Hence, there is a need for more prevalence studies outside of tropical and subtropical regions to obtain a better understanding of the zoonotic risk.

## Conclusion

The results of this systematic review and meta-analysis indicate the significant burden and current status of *S. stercoralis* infection in dogs in different parts of the world and highlight the need for studies in more geographical regions using methods with defined sensitivity. Paying attention to waste management systems, improving hygiene education and sanitary facilities in human populations as well as cleaning the environment of dog feces and establishing a preventive strategy for stray dogs could reduce the prevalence of the infection, especially in lower-income tropical and subtropical regions of the world. To decrease the burden of infective larvae in the environment contaminated with the feces of dogs, and in order to protect the canine and human population from the risk of infection, adequate deworming practices are essential. While few studies directly link infection of *Strongyloides* in humans to dogs, the shared genotypes and the similarity in where prevalence is higher support that the zoonotic potential of *S. stercoralis* infection is an important subject that should be reflected through raising awareness among dog owners and veterinarians. We recommend health authorities to organize efficient monitoring programs for protecting humans and dogs from the impact of the infection.

## Supplementary Information

**Additional file 1: References S1.** List of articles used in the meta-analysis.

**Additional file 2: Figure S1.** Sub-group analysis of the prevalence of *Strongyloides stercoralis* in included studies based on country.

**Additional file 3: Table S1.** Sub-group analysis of the prevalence of *Strongyloides stercoralis* in included studies based on continent, income level, humidity, annual rainfall, average temperature, latitude and climate.

## Data Availability

All data are included in the manuscript or as supplementary files.
