# Supplementary material for: Global prevalence and epidemiology of Strongyloides stercoralis in dogs: a systematic review and meta-analysis
Source: Parasit Vectors. 2022 Jan 10;15:21. doi: 10.1186/s13071-021-05135-0 (PMC8750836; doi:10.1186/s13071-021-05135-0)
Supplement: Supplementary file 1 — Additional file 1: References S1. List of articles used in the meta-analysis. [file 13071_2021_5135_MOESM1_ESM.docx]

**Additional file 1: References S1. List of articles used in the meta-analysis**

1. Rizzo, G. Ricciardi G. Canine stools, a poorly known factor in the problem of environmental pollution. II. Parasitological aspects. Igiene Moderna. 1978; 71 (6): 824-833
2. Ugochukwu EI, Ejimadu KN. Studies on the prevalence of gastro-intestinal helminths of dogs in Calabar, Nigeria. Int J Zoonoses. 1985;12(3):214-218.
3. Tarish JH, Al-Saqur IM, Al-Abbassy SN, Kadhim FS. The prevalence of parasitic helminths in stray dogs in the Baghdad area, Iraq. Ann Trop Med Parasitol. 1986;80(3):329-331. doi:10.1080/00034983.1986.11812024
4. Stehr-Green JK, Murray G, Schantz PM, Wahlquist SP. Intestinal parasites in pet store puppies in Atlanta. Am J Public Health. 1987;77(3):345-346. doi:10.2105/ajph.77.3.345
5. Epe C, Ising-Volmer S, Stoye M. Ergebnisse parasitologischer Kotuntersuchungen von Equiden, Hunden, Katzen und Igeln der Jahre 1984-1991 [Parasitological fecal studies of equids, dogs, cats and hedgehogs during the years 1984-1991]. Dtsch Tierarztl Wochenschr. 1993;100(11):426-428.
6. Bugg RJ, Robertson ID, Elliot AD, Thompson RC. Gastrointestinal parasites of urban dogs in Perth, Western Australia. Vet J. 1999;157(3):295-301. doi:10.1053/tvjl.1998.0327
7. Itoh N, Muraoka N, Aoki M, Itagaki T. Prevalence of *Strongyloides* spp. infection in household dogs. Kansenshogaku Zasshi. 2003;77(6):430-435. doi:10.11150/kansenshogakuzasshi1970.77.430
8. Anosike JC, Nwoke BEB, Ukaga CN, Madu NGJ, Dozie INS. Aspects of intestinal helminth parasites of dogs in World Bank-assisted housing estate, New Owerri, Nigeria. African J Applied Zoology & Environmental Biology. 2004;6:25-29.
9. Asano K, Suzuki K, Matsumoto T, Sakai T, Asano R. Prevalence of dogs with intestinal parasites in Tochigi, Japan in 1979, 1991 and 2002. Vet Parasitol. 2004;120(3):243-248. doi:10.1016/j.vetpar.2004.01.009
10. Ramı́rez-Barrios RA, Barboza-Mena G, Muñoz J, Angulo-Cubillán F, Hernández E, González F, Escalona F. Prevalence of intestinal parasites in dogs under veterinary care in Maracaibo, Venezuela. Vet Parasitol. 2004;121(1-2):11-20.
11. Komtangi MC, Mpoame M, Payne VK, Ngufor MN. Prevalence of gastrointestinal helminths of dogs in Dschang, Cameroon. J Cameroon Academy of Sciences. 2005;5(1):11-14.
12. Júnior AF, Gonçalves-Pires MR, Silva DA, Gonçalves AL, Costa-Cruz JM. Parasitological and serological diagnosis of Strongyloides stercoralis in domesticated dogs from southeastern Brazil. Vet Parasitol. 2006;136(2):137-145. doi:10.1016/j.vetpar.2005.10.022
13. Gonçalves AL, Machado GA, Gonçalves-Pires MR, Ferreira-Júnior A, Silva DA, Costa-Cruz JM. Evaluation of strongyloidiasis in kennel dogs and keepers by parasitological and serological assays. Vet Parasitol. 2007;147(1-2):132-139. doi:10.1016/j.vetpar.2007.03.016
14. Lorenzini G, Tasca T, de Carli GA. Prevalence of intestinal parasites in dogs and cats under veterinary care in Porto Alegre, Rio Grande do Sul, Brazil. Braz J Vet Res Anim Sci, Sao Paulo. 2007;44(2):137-145.
15. Papazahariadou M, Founta A, Papadopoulos E, Chliounakis S, Antoniadou-Sotiriadou K, Theodorides Y. Gastrointestinal parasites of shepherd and hunting dogs in the Serres Prefecture, Northern Greece. Vet Parasitol. 2007;148(2):170-173. doi:10.1016/j.vetpar.2007.05.013
16. Dillard KJ, Saari SAM, Anttila M. *Strongyloides stercoralis* infection in a Finnish kennel. Acta Vet Scand. 2007;49(1):37
17. Ugbomoiko US, Ariza L, Heukelbach J. Parasites of importance for human health in Nigerian dogs: high prevalence and limited knowledge of pet owners. BMC Vet Res. 2008;4:49.
18. Das SS, Kumar D, Sreekrishnan R, Ganesan R. Gastrointestinal parasitic infections in dogs in Puducherry. J Vet Parasitol. 2009;23(1):77-9.
19. Claerebout E, Casaert S, Dalemans AC, et al. Giardia and other intestinal parasites in different dog populations in Northern Belgium. Vet Parasitol. 2009;161(1-2):41-46. doi:10.1016/j.vetpar.2008.11.024
20. Gates MC, Nolan TJ. Endoparasite prevalence and recurrence across different age groups of dogs and cats. Vet Parasitol. 2009;166(1-2):153-8.
21. Itoh N, Kanai K, Hori Y, et al. Prevalence of *Giardia intestinalis* and other zoonotic intestinal parasites in private household dogs of the Hachinohe area in Aomori prefecture, Japan in 1997, 2002 and 2007. J Vet Sci. 2009;10(4):305-308.
22. Takano Y, Minakami K, Kodama S, et al. Cross infection of *Strongyloides* between humans and dogs in the Amami islands, Japan. Trop Med Health. 2009;37(4):149-152.
23. Leelayoova S, Siripattanapipong S, Naaglor T, Taamasri P, Mungthin M. Prevalence of intestinal parasitic infections in military personnel and military dogs, Thailand. J Med Assoc Thai. 2009;92 Suppl 1:S53-S59.
24. Razmi G. Survey of dogs’ parasites in Khorasan Razavi Province, Iran. Iranian J Parasitol. 2009;4(4):48-54.
25. Mariana L, Marcelina C, Teddy I, Manuel LM. Enteric parasitic in canines (*Canis familiaris*) in the urban area of Coroico, Nor yungas department of La Paz Bolivia. J Selva Andina Res Soc. 2010;1(1):37-49.
26. Zewdu E, Semahegn Y, Mekibib B. Prevalence of helminth parasites of dogs and owners awareness about zoonotic parasites in Ambo town, central Ethiopia. Ethiopian Vet J. 2010;14(2):17-30.
27. Awoke E, Bogale B, Chanie M. Intestinal nematode parasites of dogs: Prevalence and associated risk factors. Int J An Vet Advances. 2011;3(5):374-378.
28. Jones O, Kebede N, Kassa T, Tilahun G, Macias C. Prevalence of dog gastrointestinal parasites and risk perception of zoonotic infection by dog owners in Wondo Genet, Southern Ethiopia. J Public Health and Epidemiology. 2011;3(11):550-555.
29. Itoh N, Itagaki T, Kawabata T, et al. Prevalence of intestinal parasites and genotyping of Giardia intestinalis in pet shop puppies in east Japan. Vet Parasitol. 2011;176(1):74-78. doi:10.1016/j.vetpar.2010.10.048
30. Itoh N, Kanai K, Tominaga H, et al. Giardia and other intestinal parasites in dogs from veterinary clinics in Japan. Parasitol Res. 2011;109(1):253-256. doi:10.1007/s00436-011-2258-y
31. Paulos D, Addis M, Fromsa A, Mekibib B. 2012: Prevalence of gastrointestinal helminthes among dogs and owners perception about zoonotic dog parasites in Hawassa Town, Ethiopia
32. Martins CM, de Barros CdaC, Bier D, et al. Dog parasite incidence and risk factors, from sampling after one-year interval, in Pinhais, Brazil. Rev Bras Parasitol. 2012;21(2):101-106.
33. Getahun Z, Addis M. Prevalence of gastrointestinal helminthes among dogs in Bahir Dar Town, Ethiopia. World Applied Sciences Journal. 2012;19(5):595-601.
34. Mircean V, Györke A, Cozma V. Prevalence and risk factors of *Giardia duodenalis* in dogs from Romania. Vet Parasitol. 2012;184(2-4):325-329. doi:10.1016/j.vetpar.2011.08.022
35. Mekbib B, Regassa A, Sheferaw D. Gastrointestinal helminthes of dogs and owners’ perception of dogs parasitic zoonoses in Hawassa, Southern Ethiopia. J Vet Med An Hea. 2013;5(1):20-26. doi:10.5897/JVMAH12.054
36. G/selasie D, Geyola M, Dagne E, et al. Gastrointestinal helminthes in dogs and community perception on parasite zoonosis at Hawassa City, Ethiopia. Global Veterinaria. 2013;11(4):432-440.
37. Abere T, Bogale B, Melaku A.Gastrointestinal helminth parasites of pet and stray dogs as a potential risk for human health in Bahir Dar town, north-western Ethiopia. Vet World. 2013;6(7):388-392.
38. Perera PK, Rajapakse RPVJ, Rajakaruna RS. Gastrointestinal parasites of dogs in Hantana area in the Kandy District. J Natn Sci Foundation Sri Lanka. 2013;41(2):81-91.
39. Riggio F, Mannella R, Ariti G, Perrucci S. Intestinal and lung parasites in owned dogs and cats from central Italy. Vet Parasitol. 2013;193(1-3):78-84. doi:10.1016/j.vetpar.2012.11.026
40. Ortuño A, Scorza V, Castellà J, Lappin M. Prevalence of intestinal parasites in shelter and hunting dogs in Catalonia, Northeastern Spain. Vet J. 2014;199(3):465-467. doi:10.1016/j.tvjl.2013.11.022
41. Alvarado-Esquivel C, Romero-Salas D, Aguilar-Domínguez M, et al. Epidemiological assessment of intestinal parasitic infections in dogs at animal shelter in Veracruz, Mexico. Asian Pacific J Trop Biomedicine. 2015;5(1):34-39. https://doi.org/10.1016/S2221-1691(15)30167-2
42. Puebla LEJ, Nunez FA, Riveri LR, et al. Prevalence of intestinal parasites in dogs from municipality La Lisa, Havana, Cuba. J Veterinar Sci Technol. 2015;6(5):1000250. doi: 10.4172/2157-7579.1000250
43. Elom MO, Alo MN, Nworie A, et al. Ecto-and intestinal parasitic fauna of domestic dogs in two rural areas of Ebonyi State, Nigeria: Public health zoonotic jeopardy. J Entomolgy Zoology Studies. 2015;3(4):444-448.
44. Hadi AM, Faraj AA. Prevalence of gastrointestinal helminthes and protozoa among stray dogs in Baghdad. Iraqi J Vet Med. 2016;40(1):1-4.
45. Wright I, Stafford K, Coles G. The prevalence of intestinal nematodes in cats and dogs from Lancashire, north-west England. J Small Anim Pract. 2016;57(8):393-395. doi:10.1111/jsap.12478
46. Ferreira JI, Pena HF, Azevedo SS, Labruna MB, Gennari SM. Occurrences of gastrointestinal parasites in fecal samples from domestic dogs in São Paulo, SP, Brazil. Rev Bras Parasitol Vet. 2016;25(4):435-440. doi:10.1590/S1984-29612016081
47. Pumidonming W, Salman D, Gronsang D, et al. Prevalence of gastrointestinal helminth parasites of zoonotic significance in dogs and cats in lower Northern Thailand. J Vet Med Sci. 2016;78(12):1779-1784. doi:10.1292/jvms.16-0293
48. Štrkolcová G, Goldová M, Bocková E, Mojžišová J. The roundworm *Strongyloides stercoralis* in children, dogs, and soil inside and outside a segregated settlement in Eastern Slovakia: frequent but hardly detectable parasite. Parasitol Res. 2017;116(3):891-900. doi:10.1007/s00436-016-5362-1
49. Paradies P, Iarussi F, Sasanelli M, Capogna A, Lia RP, Zucca D, et al. Occurrence of strongyloidiasis in privately owned and sheltered dogs: clinical presentation and treatment outcome. Parasit Vectors. 2017;10(1):1–9.
50. Mircean V, Dumitrache MO, Mircean M, Colosi HA, Györke A. Prevalence and risk factors associated with endoparasitic infection in dogs from Transylvania (Romania): A retrospective study. Vet Parasitol. 2017;243:157-161. doi:10.1016/j.vetpar.2017.06.028
51. Jaleta TG, Zhou S, Bemm FM, Schär F, Khieu V, Muth S, et al. Different but overlapping populations of *Strongyloides stercoralis* in dogs and humans—Dogs as a possible source for zoonotic strongyloidiasis. PLoS Negl Trop Dis. 2017;11(8):e0005752
52. Sauda F, Malandrucco L, Macrì G, et al. *Leishmania infantum*, *Dirofilaria* spp. and other endoparasite infections in kennel dogs in central Italy. Leishmania infantum, Dirofilaria spp. et autres infections par les endoparasites chez les chiens de chenils d’Italie centrale. Parasite. 2018;25:2. doi:10.1051/parasite/2018001
53. García E, Gil M, Lugo M, Chacín E, Angulo-Cubillán F. Prevalence of intestinal parasites in canines from Cristo de Aranza Parish, Maracaibo Municipality, Zulia State, Venezuela. Revista Científica. 2018;28: 430-436.
54. Hurtado HAM, Forero JCG. Zoonotic parasitism in dogs from the department of Vaupés, Colombia. Braz J Hea Rev Curitiba. 2019;2(4):3408-20. doi:10.34119/bjhrv2n4-103
55. Iatta R, Buonfrate D, Paradies P, et al. Occurrence, diagnosis and follow-up of canine strongyloidiosis in naturally infected shelter dogs. Parasitology. 2019;146(2):246-252. doi:10.1017/S0031182018001312
56. Sanchez-Thevenet P, Carmena D, Adell-Aledón M, et al. High prevalence and diversity of zoonotic and other intestinal parasites in dogs from eastern Spain. Vector Borne Zoonotic Dis. 2019;19(12):915-922. doi:10.1089/vbz.2019.2468
57. Kurnosova OP, Arisov MV, Odoyevskaya IM. Intestinal parasites of pets and other house-kept animals in Moscow. Helminthologia. 2019;56(2):108-117. doi: 10.2478/helm-2019-0007
58. Sanpool O, Intapan PM, Rodpai R, et al. Dogs are reservoir hosts for possible transmission of human strongyloidiasis in Thailand: molecular identification and genetic diversity of causative parasite species. J Helminthol. 2019;94:e110. doi:10.1017/S0022149X1900107X
59. Beknazarova M, Whiley H, Traub R, Ross K. Opportunistic mapping of *Strongyloides stercoralis* and hookworm in dogs in remote Australian communities. Pathogens. 2020;9(5):398. doi:10.3390/pathogens9050398
60. Dashchenko S, Soroka N, Semenko O. Distribution of *Strongyloides stercoralis* among dogs of different housing groups in Kyiv and Kyiv region, clinical manifestations and diagnostic methods. Vet Sci Vet Med. 2020;5. https://doi.org/10.21303/2504-5679.2020.001423
61. Nagamori Y, Payton ME, Looper E, Apple H, Johnson EM. Retrospective survey of endoparasitism identified in feces of client-owned dogs in North America from 2007 through 2018. Vet Parasitol. 2020;282:109137. doi:10.1016/j.vetpar.2020.109137
