# Supplementary material for: Global prevalence and epidemiology of Strongyloides stercoralis in dogs: a systematic review and meta-analysis
Source: Parasit Vectors. 2022 Jan 10;15:21. doi: 10.1186/s13071-021-05135-0 (PMC8750836; doi:10.1186/s13071-021-05135-0)

Additional file 2: Figure S1. Sub-group analysis of the prevalence of *Strongyloides stercoralis* in included studies based on country.

Study	Prevalence	95% CI
**Australia**		
M. Beknazarova 2020 Australia	0.2190	[0.1741; 0.2716]
R.J. Bugg 1999 Australia	0.0024	[0.0004; 0.0133]
**Random effects model**	**0.0699**	**[0.0000; 1.0000]**
Heterogeneity: $I^2 = 99.2156\%$, $\tau^2 = 0.0945$, $p < .001$		
**Belgium**		
E. Claerebout 2009 Belgium	0.0009	[0.0002; 0.0049]
**Random effects model**	**0.0009**	**[0.0000; 0.0034]**
Heterogeneity: not applicable		
**Bolivia**		
L. Mariana 2010 Bolivia	0.0417	[0.0163; 0.1023]
**Random effects model**	**0.0417**	**[0.0111; 0.0905]**
Heterogeneity: not applicable		
**Brazil**		
A. F. Junior 2006 Brazil	0.0093	[0.0026; 0.0333]
A.L.R. Goncalves 2007 Brazil	0.0055	[0.0010; 0.0306]
C.M. Martins 2012 Brazil	0.2632	[0.2029; 0.3338]
G. Lorenzini 2007 Brazil	0.0041	[0.0019; 0.0089]
J.I.G.S. Ferreira 2016 Brazil	0.0010	[0.0003; 0.0028]
**Random effects model**	**0.0252**	**[0.0000; 0.1678]**
Heterogeneity: $I^2 = 97.6246\%$, $\tau^2 = 0.0436$, p = NA		
**Cambodia**		
T.G. Jaleta 2017 Cambodia	0.8547	[0.7796; 0.9073]
**Random effects model**	**0.8547**	**[0.7854; 0.9124]**
Heterogeneity: not applicable		
**Cameroon**		
M.C. Komtangi 2005 Cameroon	0.1145	[0.0706; 0.1803]
**Random effects model**	**0.1145**	**[0.0659; 0.1744]**
Heterogeneity: not applicable		
**Colombia**		
H.A.M. Hurtado 2019 Colombia	0.0546	[0.0367; 0.0806]
**Random effects model**	**0.0546**	**[0.0350; 0.0783]**
Heterogeneity: not applicable		
**Cuba**		
L.E.J. Puebla 2015 Cuba	0.0206	[0.0057; 0.0721]
**Random effects model**	**0.0206**	**[0.0020; 0.0582]**
Heterogeneity: not applicable		
**Ethiopia**		
B. Mekbib 2013 Ethiopia	0.6012	[0.5681; 0.6334]
D. G. selasie 2013 Ethiopia	0.3058	[0.2649; 0.3500]
D. Paulos 2012 Ethiopia	0.5802	[0.5344; 0.6247]
E. Awoke 2011 Ethiopia	0.0429	[0.0258; 0.0708]
E. Zewdu 2010 Ethiopia	0.2541	[0.1852; 0.3380]
O. Jones 2011 Ethiopia	0.4610	[0.4024; 0.5207]
T. Abere 2013 Ethiopia	0.2372	[0.1994; 0.2796]
Z. Getahun 2012 Ethiopia	0.2161	[0.1779; 0.2600]
**Random effects model**	**0.3211**	**[0.1617; 0.5056]**
Heterogeneity: $I^2 = 98.8952\%$, $\tau^2 = 0.0500$, $p = .97$		
**Finland**		
K.J. Dillard 2007 Finland	0.0652	[0.0224; 0.1750]
**Random effects model**	**0.0652**	**[0.0129; 0.1536]**
Heterogeneity: not applicable		
**Germany**		
C. Epe 1993 Germany	0.0030	[0.0016; 0.0055]
**Random effects model**	**0.0030**	**[0.0014; 0.0051]**
Heterogeneity: not applicable		
**Greece**		
M. Papazahariadou 2007 Greece	0.0178	[0.0076; 0.0410]
**Random effects model**	**0.0178**	**[0.0057; 0.0365]**
Heterogeneity: not applicable		
**India**		
S.S. Das 2009 India	0.0429	[0.0210; 0.0860]
**Random effects model**	**0.0429**	**[0.0173; 0.0793]**
Heterogeneity: not applicable		
**Iran**		
Gh.R. Razmi 2009 Iran	0.0287	[0.0123; 0.0655]
**Random effects model**	**0.0287**	**[0.0092; 0.0587]**
Heterogeneity: not applicable		
**Iraq**		
A.M. Hadi 2016 Iraq	0.0500	[0.0231; 0.1048]
J.H. Tarish 1986 Iraq	0.0500	[0.0089; 0.2361]
**Random effects model**	**0.0500**	**[0.0500; 0.0500]**
Heterogeneity: $I^2 = 0\%$, $\tau^2 = 0$, $p < .001$		
**Italy**		
F. Riggio 2013 Italy	0.0084	[0.0023; 0.0300]
F. Sauda 2018 Italy	0.0016	[0.0003; 0.0088]
G. Rizzo 1978 Italy	0.0059	[0.0010; 0.0326]
P. Paradies 2017 Italy	0.0221	[0.0101; 0.0473]
R. Iatta 2019 Italy	0.3600	[0.2727; 0.4576]
**Random effects model**	**0.0386**	**[0.0000; 0.2360]**
Heterogeneity: $I^2 = 96.9002\%$, $\tau^2 = 0.0604$, p = NA		
**Japan**		
K. Asano 2004 Japan	0.0104	[0.0053; 0.0203]
N. Itoh 2003 Japan	0.0186	[0.0129; 0.0268]
N. Itoh 2011 Japan	0.0111	[0.0072; 0.0172]
N. Itoh 2011 Japan	0.0025	[0.0012; 0.0055]
N. Itoh 2009 Japan	0.0109	[0.0062; 0.0189]
Y. Takano 2009 Japan	0.0909	[0.0395; 0.1958]
**Random effects model**	**0.0144**	**[0.0018; 0.0387]**
Heterogeneity: $I^2 = 87.6675\%$, $\tau^2 = 0.0053$, p = NA		
**Mexico**		
C. Alvarado-Esquivel 2015 Mexico	0.1584	[0.0999; 0.2419]
**Random effects model**	**0.1584**	**[0.0941; 0.2356]**
Heterogeneity: not applicable		
**Nigeria**		
E.I. Ugochukwu 1985 Nigeria	0.1496	[0.1110; 0.1987]
J.C. Anosike 2004 Nigeria	0.1065	[0.0835; 0.1349]
M.O. Elom 2015 Nigeria	0.0300	[0.0103; 0.0845]
U.S. Ugbomoiko 2008 Nigeria	0.0379	[0.0231; 0.0615]
**Random effects model**	**0.0753**	**[0.0117; 0.1874]**
Heterogeneity: $I^2 = 91.4411\%$, $\tau^2 = 0.0101$, p = NA		
**Romania**		
V. Mircean 2012 Romania	0.0385	[0.0106; 0.1298]
V. Mircean 2017 Romania	0.0350	[0.0263; 0.0464]
**Random effects model**	**0.0351**	**[0.0274; 0.0438]**
Heterogeneity: $I^2 = 0\%$, $\tau^2 = < 0.0001$, $p = .08$		
**Russia**		
O. P. Kurnosova 2019 Russia	0.0091	[0.0056; 0.0148]
**Random effects model**	**0.0091**	**[0.0052; 0.0141]**
Heterogeneity: not applicable		
**Slovakia**		
G. Strkolcova 2017 Slovakia	0.1200	[0.0562; 0.2380]
**Random effects model**	**0.1200**	**[0.0456; 0.2234]**
Heterogeneity: not applicable		
**Spain**		
A. Ortuno 2014 Spain	0.0118	[0.0033; 0.0421]
P. Sanchez-Thevenet 2019 Spain	0.0114	[0.0039; 0.0330]
**Random effects model**	**0.0116**	**[0.0091; 0.0144]**
Heterogeneity: $I^2 = 0\%$, $\tau^2 = < 0.0001$, p = NA		
**Sri Lanka**		
P.K. Perera 2013 Sri Lanka	0.1111	[0.0615; 0.1926]
**Random effects model**	**0.1111**	**[0.0549; 0.1838]**
Heterogeneity: not applicable		
**Thailand**		
O. Sanpool 2020 Thailand	0.0417	[0.0179; 0.0938]
S.Leelayoova 2009 Thailand	0.0053	[0.0009; 0.0294]
W. Pumidonming 2016 Thailand	0.0152	[0.0052; 0.0438]
**Random effects model**	**0.0168**	**[0.0000; 0.0831]**
Heterogeneity: $I^2 = 61.3545\%$, $\tau^2 = 0.0028$, p = NA		
**UK**		
I. Wright 2016 UK	0.0175	[0.0060; 0.0503]
**Random effects model**	**0.0175**	**[0.0033; 0.0426]**
Heterogeneity: not applicable		
**Ukraine**		
S. Dashchenko 2020 Ukraine	0.0275	[0.0150; 0.0498]
**Random effects model**	**0.0275**	**[0.0132; 0.0467]**
Heterogeneity: not applicable		
**USA**		
J.K. Stehr-Green 1987 USA	0.0140	[0.0038; 0.0496]
M.C. Gates 2009 USA	0.0020	[0.0012; 0.0034]
Y. Nagamori 2020 USA	0.0020	[0.0012; 0.0033]
**Random effects model**	**0.0031**	**[0.0000; 0.0185]**
Heterogeneity: $I^2 = 35.1132\%$, $\tau^2 = 0.0009$, $p < .001$		
**Venezuela**		
E. García 2018 Venezuela	0.0163	[0.0070; 0.0377]
R.A. Ramirez-Barrios 2004 Venezuela	0.0033	[0.0009; 0.0118]
**Random effects model**	**0.0080**	**[0.0000; 0.2637]**
Heterogeneity: $I^2 = 75.7666\%$, $\tau^2 = 0.0017$, $p < .001$		
**Random effects model**	**0.0618**	**[0.0356; 0.0945]**
Heterogeneity: $I^2 = 99.0421\%$, $\tau^2 = 0.0558$, $p < .001$		
Test for subgroup differences: $\chi^2_{28} = 2430.9232$, df = 28 ($p < .001$)		

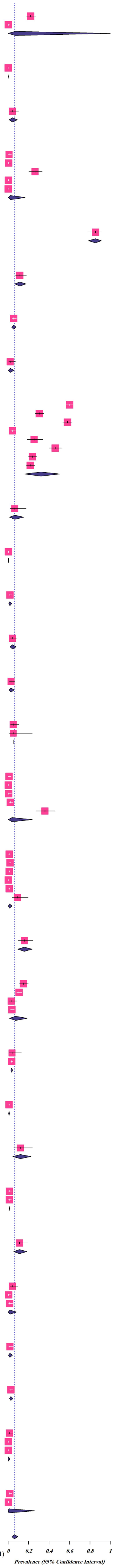

Supplement: Supplementary file 2 — Additional file 2: Figure S1. Sub-group analysis of the prevalence of Strongyloides stercoralis in included studies based on country. [file 13071_2021_5135_MOESM2_ESM.pdf]
