# Supplementary material for: Global prevalence and epidemiology of Strongyloides stercoralis in dogs: a systematic review and meta-analysis
Source: Parasit Vectors. 2022 Jan 10;15:21. doi: 10.1186/s13071-021-05135-0 (PMC8750836; doi:10.1186/s13071-021-05135-0)
Supplement: Supplementary file 3 — Additional file 3: Table S1. Sub-group analysis of the prevalence of Strongyloides stercoralis in included studies based on continent, income level, humidity, annual rainfall, average temperature, latitude and climate. [file 13071_2021_5135_MOESM3_ESM.docx]

**Additional file 3: Table S1.** Sub-group analysis of the prevalence of *Strongyloides stercoralis* in included studies based on continent, income level, humidity, annual rainfall, average temperature, latitude, and climate.

| Variable | Number of studies | | Sample size | Infected | Pooled prevalence  (95% CI) | Heterogeneity | | |
| --- | --- | --- | --- | --- | --- | --- | --- | --- |
|  |  |  |  |  |  | **I^2^** | **τ^2^** | ***P*-value** |
|  | | | | | | | | |
| **Continent** | |  | | | | | | |
| Oceania | | 2 | 695 | 61 | 6 % (0 % - 100%) | 99 | 0.094 | < 0.01 |
| North America | | 5 | 14305 | 48 | 2 % (0 % - 10%) | 93 | 0.020 | < 0.01 |
| South America | | 9 | 6576 | 91 | 2 % (0 % - 7%) | 96 | 0.023 | < 0.01 |
| Europe | | 17 | 10370 | 153 | 2 % (0 % - 5%) | 93 | 0.019 | < 0.01 |
| Asia | | 15 | 8786 | 217 | 5 % (0 % - 14%) | 97 | 0.072 | < 0.01 |
| Africa | | 13 | 4729 | 1402 | 21 % (10 % -34%) | 98 | 0.057 | < 0.01 |
| **Income level** | |  |  |  |  |  |  |  |
| High | | 28 | 32768 | 323 | 2 % (0 % - 4%) | 95 | 0.018 | < 0.01 |
| Upper middle | | 14 | 7358 | 119 | 2 % (0 % - 6%) | 95 | 0.019 | < 0.01 |
| Lower middle | | 7 | 737 | 143 | 14 % (0 % - 43%) | 98 | 0.122 | < 0.01 |
| Low | | 12 | 4598 | 1387 | 22 % (10% - 36%) | 99 | 0.060 | < 0.01 |
| **Humidity (%)** | |  | | | | | | |
| <40 | | 3 | 314 | 12 | 3 % (1% - 7%) | 97 | 0 | < 0.01 |
| 40-75 | | 41 | 30697 | 1768 | 8 % (4% - 13%) | 99 | 0.072 | < 0.01 |
| >75 | | 17 | 14450 | 192 | 2 % (1% - 5%) | 95 | 0.017 | < 0.01 |
| **Annual rainfall (mm)** | |  | | | | | | |
| <400 | | 4 | 595 | 17 | 2 % (1% - 5%) | 5 | 0 | < 0.01 |
| 401-1000 | | 20 | 11044 | 227 | 3 % (1% - 6%) | 95 | 0.0225 | < 0.01 |
| 1001-1500 | | 33 | 32681 | 1705 | 9 % (4% - 15%) | 99 | 0.083 | < 0.01 |
| >1500 | | 4 | 1141 | 32 | 4 % (0% - 18%) | 94 | 0.020 | < 0.01 |
| **Average temperature (°C)** | |  | | | | | | |
| <10 | | 3 | 2162 | 29 | 2 % (0% - 10%) | 80 | 0.004 | < 0.01 |
| 10-20 | | 37 | 34692 | 1623 | 6 % (3% - 11%) | 99 | 0.058 | < 0.01 |
| >20 | | 21 | 8607 | 329 | 5 % (1% - 12%) | 98 | 0.060 | < 0.01 |
| **Latitude** | |  | | | | | | |
| 1-25° | | 29 | 12216 | 1630 | 11 % (5% - 19%) | 99 | 0.082 | < 0.01 |
| 26-40° | | 16 | 23156 | 194 | 2 % (0% - 4%) | 95 | 0.012 | < 0.01 |
| 41-60° | | 16 | 10089 | 148 | 2 % (0% - 5%) | 93 | 0.020 | < 0.01 |
| **Climate** | |  |  |  |  |  |  |  |
| Subpolar climate | | 1 | 46 | 3 | 6 % (1% - 15%) | - | - | - |
| Marine climate | | 4 | 1762 | 9 | 0 % (0% - 2%) | 74 | 0.001 | < 0.01 |
| Humid subtropical climate | | 13 | 9711 | 186 | 3 % (0% - 8%) | 96 | 0.033 | < 0.01 |
| Tropical wet and dry climate | | 25 | 11508 | 1577 | 12 % (5% - 21%) | 99 | 0.094 | < 0.01 |
| Tropical rainforest climate | | 2 | 511 | 33 | 7 % (0% - 63%) | 68 | 0.003 | 0.07 |
| Warm humid continental climate | | 9 | 20968 | 120 | 1 % (0% - 3%) | 94 | 0.007 | < 0.01 |
| Hot-summer Mediterranean climate | | 2 | 455 | 10 | 2 % (0% - 13%) | 0 | 0 | 0.08 |
| Cold semi-desert climate | | 1 | 96 | 4 | 4 % (1% - 9%) | - | - | - |
| Hot semi-desert climates | | 2 | 264 | 23 | 9 % (0% - 100%) | 90 | 0.016 | < 0.01 |
| Hot desert climates | | 2 | 140 | 7 | 5 % (5% - 5%) | 0 | 0 | < 0.01 |
